# Biological modelling of the radiation dose escalation effect of regional hyperthermia in cervical cancer

**DOI:** 10.1186/s13014-016-0592-z

**Published:** 2016-02-02

**Authors:** J. Crezee, C. M. van Leeuwen, A. L. Oei, L. E. van Heerden, A. Bel, L. J. A. Stalpers, P. Ghadjar, N. A. P. Franken, H. P. Kok

**Affiliations:** Department of Radiation Oncology, Academic Medical Center, University of Amsterdam, Meibergdreef 9, 1105 AZ Amsterdam, The Netherlands; Laboratory for Experimental Oncology and Radiobiology (LEXOR)/Center for Experimental and Molecular Medicine, Meibergdreef 9, 1105 AZ Amsterdam, The Netherlands; Department of Radiation Oncology, Charité Universitätsmedizin Berlin, Augustenburger Platz 1, Berlin, 13353 Germany

**Keywords:** Hyperthermia, Radiotherapy, Linear-quadratic model, Treatment planning

## Abstract

**Background:**

Locoregional hyperthermia combined with radiotherapy significantly improves locoregional control and overall survival for cervical tumors compared to radiotherapy alone. In this study biological modelling is applied to quantify the effect of radiosensitization for three cervical cancer patients to evaluate the improvement in equivalent dose for the combination treatment with radiotherapy and hyperthermia.

**Methods:**

The Linear-Quadratic (LQ) model extended with temperature-dependent LQ-parameters *α* and *β* was used to model radiosensitization by hyperthermia and to calculate the conventional radiation dose that is equivalent in biological effect to the combined radiotherapy and hyperthermia treatment. External beam radiotherapy planning was performed based on a prescription dose of 46Gy in 23 fractions of 2Gy. Hyperthermia treatment using the AMC-4 system was simulated based on the actual optimized system settings used during treatment.

**Results:**

The simulated hyperthermia treatments for the 3 patients yielded a T50 of 40.1 °C, 40.5 °C, 41.1 °C and a T90 of 39.2 °C, 39.7 °C, 40.4 °C, respectively. The combined radiotherapy and hyperthermia treatment resulted in a D95 of 52.5Gy, 55.5Gy, 56.9Gy in the GTV, a dose escalation of 7.3–11.9Gy compared to radiotherapy alone (D95 = 45.0–45.5Gy).

**Conclusions:**

This study applied biological modelling to evaluate radiosensitization by hyperthermia as a radiation-dose escalation for cervical cancer patients. This model is very useful to compare the effectiveness of different treatment schedules for combined radiotherapy and hyperthermia treatments and to guide the design of clinical studies on dose escalation using hyperthermia in a multi-modality setting.

## Background

Achieving locoregional control is a challenge for locally advanced cervix tumors (FIGO stage IIB-IV). Currently combination of radiotherapy with concurrent cisplatin-based chemotherapy is widely accepted as a standard treatment [1–5]. An alternative is combining radiotherapy with hyperthermia. Hyperthermia (i.e. increasing tumor temperatures to 40–45 °C) is a very powerful radio and chemosensitizer [6–9] improving clinical outcome [10–12]. Several randomized clinical trials have demonstrated that locoregional hyperthermia combined with radiotherapy (thermoradiotherapy) is at least as effective in improving locoregional control for cervical tumors as chemoradiotherapy [13–17]. The Dutch Deep Hyperthermia Trial not only showed an 83 % local control rate for radiotherapy + hyperthermia versus 57 % for radiotherapy alone, but also a 3-year overall survival of 51 % for radiotherapy combined with hyperthermia, compared to 27 % with radiotherapy alone [13]. After 12 years survival is still almost twice as high after treatment with thermoradiotherapy (37 %), versus 20 % with radiotherapy [18]. A major advantage of hyperthermia given sequentially before or after radiotherapy is that the incidence of late toxicity grade 3 or higher is not significantly different from the toxicity reported after treatment with radiotherapy alone. Thermoradiotherapy is therefore often used as an alternative for the large number of stage IIB-IV patients unable to receive the standard therapy of concurrent chemotherapy and radiotherapy, e.g. because of kidney failure. Local tumor control achieved for standard therapy is good, particularly if combined with image guided brachytherapy [19–22], the challenge is still to improve control of regional and distant disease and the reduction of side effects. Hyperthermia may be effective in achieving these objectives.

Planning for locoregional hyperthermia has become a standard clinical procedure [23–25] but does not take the synergy with radiotherapy into account. The radiosensitizing effect of hyperthermia can be considered as a local increase in tumor dose, which can also be quantified using biological modelling [26, 27]. Hyperthermic radiosensitization can be modelled as a change in the α and ß parameters of the Linear Quadratic model [28–30]. Recently a method has been presented to estimate the effect of a hyperthermia treatment in terms of equivalent dose distributions, i.e. the radiation dose that has a biological effect equivalent to that of the combined hyperthermia plus radiotherapy treatment. A conservative estimate of the temperature-dependency of the modelling parameters was applied due to lack of published temperature-dependent radiosensitivity data [31].

In this study biological modelling was applied for three cervical cancer patients treated with radiotherapy (external beam + brachytherapy) and hyperthermia to evaluate the improvement in equivalent dose for this combined treatment. The radiotherapy dose distribution was matched onto the geometry of the hyperthermia data set. Temperature-dependent parameters describing the radiosensitivity were estimated from in vitro experiments with a human cervical cancer cell line [29]. Hyperthermia treatments were simulated to evaluate the difference in equivalent dose. Predicted temperature distributions were compared to the measured tumor temperatures.

## Methods

**Fig. 1:**
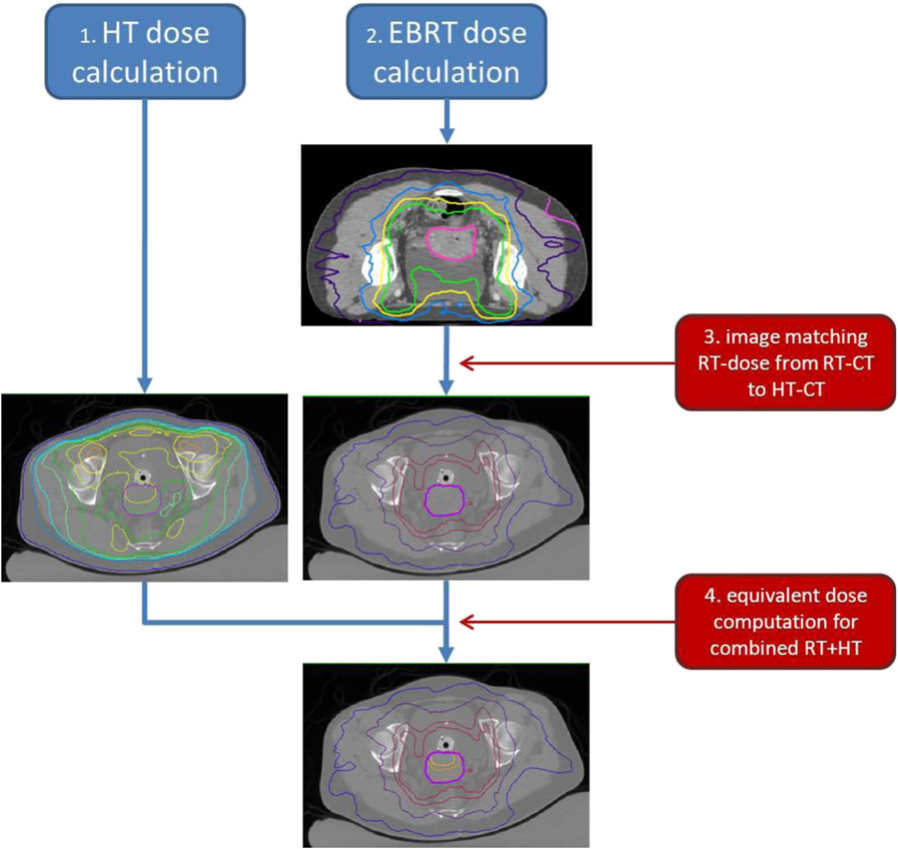
Workflow. The workflow used consists of first computation of the hyperthermia and radiotherapy dose distributions, followed by matching the radiotherapy dose distribution onto the geometry of the hyperthermia CT scan and finally computation of the equivalent dose distribution

The workflow used consists of first computation of the hyperthermia and radiotherapy dose distributions followed by matching the radiotherapy dose distribution onto the geometry of the hyperthermia CT scan and finally computation of the equivalent dose distribution (Fig. 1).

### Hyperthermia treatment planning

Hyperthermia treatment planning [25] is a standard clinical procedure at the AMC and is based on a CT-scan in hyperthermia treatment position, i.e. in supine position on a water bolus and mattresses. Treatment planning was performed using a non-commercial software package developed at the AMC. Hounsfield Unit based segmentation was applied to segment the CT scan into muscle, fat, bone and air [32], to which literature-based dielectric and thermal tissue properties were assigned (Table 1). The tumor was delineated manually by a radiation oncologist. Hyperthermia treatment was simulated for the AMC-4 locoregional heating device. Electric field distributions in the patient were calculated at a resolution of 2.5x2.5x2.5 mm^3^ by solving Maxwell’s equations with the Finite Difference Time Domain method [33]. Antenna settings yielding an optimal steady-state tumor temperature distribution were determined using temperature-based optimization [34]. Heat-transfer computations for perfused tissues were based on the Pennes bio heat model [35]. In clinical hyperthermia tumor temperatures are usually reported as *T10*, *T50* and *T90*, i.e. the temperature at least achieved in 10, 50 and 90 % of the target volume, respectively [36, 37]. A tumor temperature of 43 °C was the objective for optimization, normal tissue temperatures were constrained to 45 °C.

**Table 1:** Dielectric and thermal tissue properties used in hyperthermia treatment planning

Tissue type	Conductivity σ	Relative permittivity ε_r_	Density *ρ*	Perfusion *W*_*b*_	Conductivity *k*	Capacity c
	[S m^-1^]		[kg m^-3^]	[kg m^-3^ s^-1^]	[W m^-1^ K^-1^]	[J kg^-1^ K^-1^]
Air	0	1	1.29	0	0.024	10,000^a^
Bone	0.05	10	1595	0.12	0.65	1420
Muscle	0.75	75	1050	3.6	0.56	3639
Fat	0.06	10	888	1.1	0.217	2387
Cervical tumor	0.74	65	1050	1.8	0.56	3639

^a^The value of c used for air was tenfold increased to accelerate thermal computations. This has a negligible effect on the steady-state temperature (<2 × 10^−5^ °C)

Subsequently five weekly treatment sessions were given during the period external beam irradiation was given, one hour after a radiotherapy session. Hyperthermia was performed with the AMC-4 system consisting of four 70 MHz waveguides around the pelvis of the patient, with water bags cooled to 13 °C between waveguide and skin provide coupling of energy and skin cooling [38]. Thermometry consisted of thermocouple thermometry probes in the bladder, rectum and in a vaginal pelotte for temperature measurements during hyperthermia, yielding measurement points bordering the GTV. Phase settings during treatment are the pre-planned settings, modified after performing phase sweeps at the start of treatment [36], followed by a final phase optimization using three delta T pulses to achieve preferential heating of the cervix compared to bladder and rectum [39]. The simulated tumor temperatures for these phase and amplitude settings were used for biological modelling and were also compared with temperatures measured during the first hyperthermia session. The first session was chosen since the patient anatomy during this session best matches the anatomy as determined during CT and are thus expected to yield more reliable planning results than for later sessions.

### Radiotherapy treatment planning

Radiotherapy treatment for locally advanced cervical cancer consists of a combination of external beam and MRI-guided brachytherapy. The prescription dose for external beam irradiation was 46Gy in 23 fractions of 2Gy. External beam radiotherapy planning was performed using Oncentra and based on a CT scan in prone position on a belly board. External beam radiotherapy was followed by a 24Gy brachytherapy boost using PDR brachytherapy. Brachytherapy planning is based on an MRI scan with the applicator in situ. The hyperthermia treatment is unlikely to enhance the brachytherapy dose since hyperthermia is only given during external beam radiotherapy (EBRT) at the AMC and since there is a gap of one week between the EBRT and brachytherapy treatments. Thus we performed our evaluation for the combination of hyperthermia and EBRT without involving the brachytherapy boost.

### Image registration/matching

**Fig. 2:**
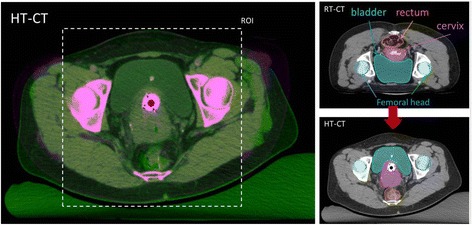
Image registration/matching. Radiotherapy and hyperthermia CT scans are rigidly matched by visual assessment of the bony anatomy, followed by deformable registration using intensity-based deformable image registration software of Velocity Medical Solutions (Varian Medical systems, Palo Alto). Left: overlay showing the excellent match of the RT-CT onto the HT-CT in the Region of Interest (ROI) indicated with the dotted rectangle. Right: Outlines of rectum, cervix, bladder and part of the bony anatomy are shown in the RT-CT and the HT-CT

After treatment planning for both modalities, the next step is matching the radiotherapy dose distribution onto the geometry of the hyperthermia data set. To account for organ displacement and deformation due to different patient positioning (prone/supine) in the radiotherapy and hyperthermia CT scans, deformable matching software of Velocity Medical Solutions (Varian Medical systems, Palo Alto) was used (Fig. 2). First, the radiotherapy and hyperthermia CT scans were rigidly matched by visual assessment of the bony anatomy. Next, a deformable registration was made between the EBRT CT and the hyperthermia CT using an intensity-based deformable image registration algorithm. As this study focusses on the effect of hyperthermia on the tumor, accuracy of the deformable match was assessed by warping contours of organs located close to the GTV from the EBRT CT to the hyperthermia CT and verifying that their location was sufficiently accurate. If not, these organs were delineated on the hyperthermia scan as well and a new deformable image registration was performed (starting again from the rigid match), this time using a hybrid algorithm combining both intensity and structure based matching. The resulting deformation vector field was used to warp the radiotherapy dose distribution to the frame of reference of the hyperthermia CT.

### Biological modelling

To quantify the therapeutic effect of radiosensitization by hyperthermia, the radiotherapy dose distributions combined with hyperthermia were converted to equivalent radiation dose distributions (without hyperthermia in 2Gy/fraction (EQD_2_)). Calculation of equivalent radiation dose was based on the LQ-model [27], extended with temperature-dependent LQ-parameters *α(T)* and *β(T)* derived for cervical cancer cells in the hyperthermic temperature range as is described in the next paragraph.

### LQ-parameters

Franken et al [29] reported α and β values for a human cervical cancer cell line (SiHa) at 37, 41 and 43 °C listed in Table 2. These values were used to define the temperature-dependent LQ-parameters *α(T)* and *β(T)* for the entire hyperthermic range needed for the LQ-model. The most significant change observed between 37 and 41 °C is an increase in *β*, whereas *α* increases strongly between 41 and 43 °C. We therefore applied a piecewise-linear interpolation for *α(T)* and *β(T)*, consisting of one linear segment valid between 37 and 41 °C and another linear segment valid between 41 and 43 °C. To evaluate the impact of using realistic temperature-dependent LQ-parameters the outcome predicted by the radiobiological model using this piecewise-linear interpolation was then compared to the outcome using a linear interpolation using only the data for *α* and *β* at 37 and 43 °C.

**Table 2:** LQ-parameters α and β used in the equivalent dose calculations

Temperature [°C]	α [Gy^-1^]	β [Gy^-2^]
37	0.33 ± 0.06	0.02 ± 0.01
41	0.31 ± 0.05	0.09 ± 0.02
43	0.76 ± 0.04	0.09 ± 0.01

α and β values derived from clonogenic assays of SiHa cervical tumor cells subjected to 1 h of hyperthermia treatment at 37, 41 or 43 °C [29]

### Equivalent dose calculations

Equivalent dose distributions for combined radiotherapy-hyperthermia treatments were calculated using the LQ-model [27]. The expression used for EQD_2_ calculation is:

$$ EQ{D}_2=\frac{\alpha (T)\cdot D+\beta (T)\cdot d\cdot D}{\alpha (37)+\beta (37)\cdot 2} $$

with fraction dose *d* and total dose *D* and using the temperature-dependent LQ-parameters *α* and *β* as described in the previous section on LQ-parameters. Most of the procedure is similar to the procedure used in the simulation study of Kok et al. for prostate cancer [31]. Hyperthermic radiosensitization is assumed to be tumor selective since radiotherapy and hyperthermia are given sequentially with a time interval of at least 1 h [40–42]. The temperature-dependent LQ-parameters *α* and *β* were thus only applied within the Gross Tumor Volume (GTV), elsewhere *α* and *β* were assumed to remain unchanged.

## Results

### Temperature and Dose Volume Histogram

**Fig. 3:**
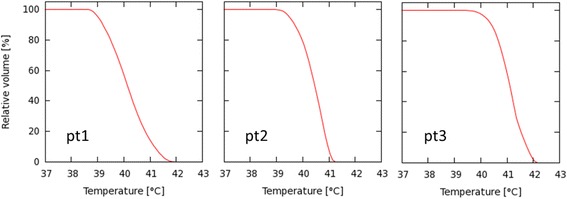
Temperature Volume Histogram. Temperature Volume Histogram (TVH) representing the simulated temperature distribution within the GTV for patient 1, 2 and 3

**Fig. 4:**
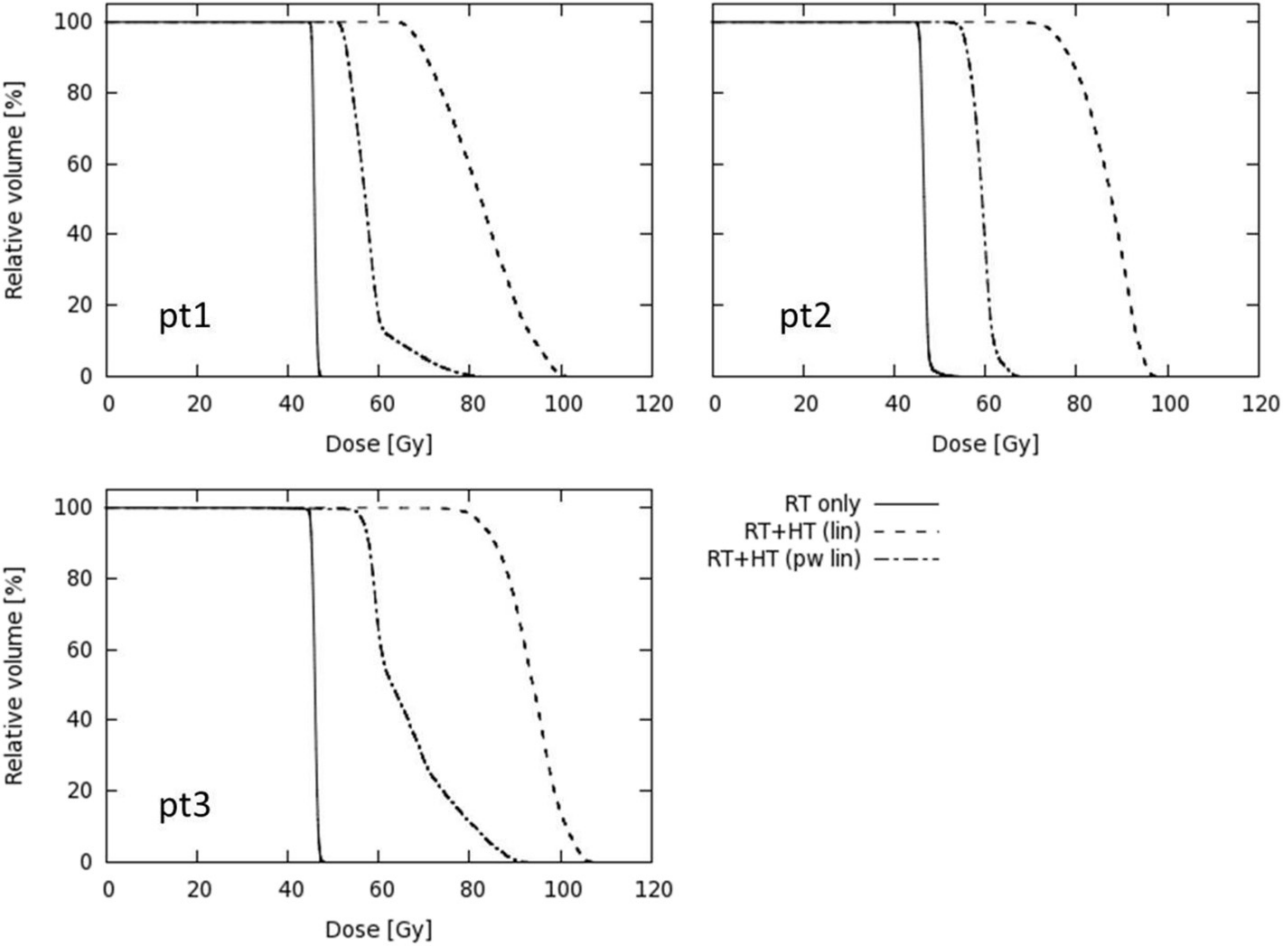
Dose Volume Histogram. Dose Volume Histogram reflecting the radiotherapy dose distribution within the GTV for patient 1, 2 and 3 comparing 3 different cases: Radiotherapy alone (RT only), radiotherapy and hyperthermia using a linear interpolation (RT + HT lin) or a piecewise linear interpolation (RT + HT pw lin) to the α and β values from Franken et al. [29]. EBRT only, brachytherapy boost not taken into account

The temperature volume histograms indicating the tumor temperature distribution are shown in Fig. 3. The corresponding *T10*, *T50* and *T90* values are listed in Table 3 as well as the temperature measured during the first treatment session. The effect of adding hyperthermia is shown in the cumulative Dose Volume Histograms (DVH) in Fig. 4. These are computed assuming either a linear or a piecewise-linear temperature dependency for *α(T)* and *β(T)*, yielding a remarkable difference in predicted dose escalation with a much more pronounced effect for the linear temperature dependency: D95 ranges between 68.4Gy and 82.9Gy for the linear interpolation compared to 52.5–56.9Gy for the piecewise linear interpolation and 45.0-45.5Gy for radiotherapy alone (Table 3). The piecewise linear interpolation results in a bending point in the DVH, separating the sections of the curve representing temperatures lower or higher than 41 °C. Temperatures higher than 41 °C result in far more significant dose escalation than temperatures below 41 °C.

**Table 3:** Radiotherapy and hyperthermia dose distribution

Pt#	Simulated T90 [°C]	T50 [°C]	T10 [°C]	D95 RT [Gy]	RT + HT (lin)	RT + HT (pw-lin)	Measured T90 [°C]	T50 [°C]	T10 [°C]
1	39.2	40.1	41.2	45.2	68.4	52.5	39.5	40.0	40.5
2	39.7	40.5	41.0	45.5	76.2	55.5	39.3	40.1	40.8
3	40.4	41.1	41.8	45.0	82.9	56.9	40.5	41.4	42.2

Simulation of D95, T10, T50 and T90 in GTV and measurement of T10, T50 and T90 for patients 1 to 3

### Equivalent Dose distributions

**Fig. 5:**
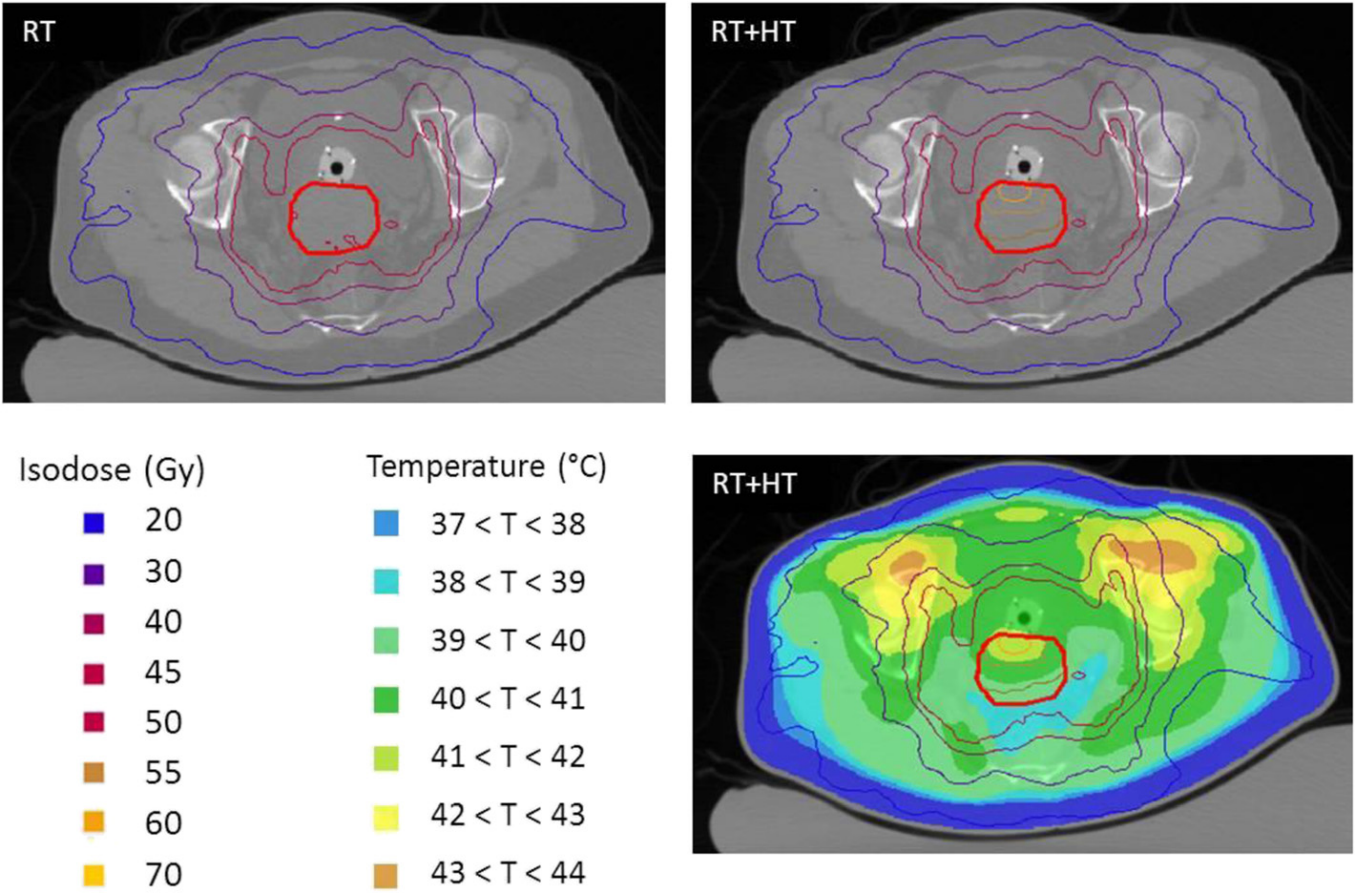
Radiotherapy and hyperthermia dose distributions. Radiotherapy isodose curves (top left), equivalent radiotherapy isodose curves for radiotherapy + hyperthermia (top right), idem with the hyperthermia temperature distribution overlaid as a color wash (bottom right) for patient #1 with cervical cancer. Radiotherapy isodose curves overlaid on the CT scan of the patient made in hyperthermia position lying on a hyperthermia water bolus. Gross tumour volume (GTV) is indicated by the bold red contour. The contribution of hyperthermia to equivalent radiotherapy isodose is visible within the GTV and increases with increasing temperature in ventral direction. Vaginal pelotte for temperature measurements during hyperthermia is visible adjacent to GTV

Radiotherapy dose distributions for patient 1 with and without hyperthermia are shown in Fig. 5. Temperature sensitive LQ-parameters *α(T)* and *β(T)* were applied to the GTV using the piecewise-linear interpolation to the LQ-data listed in Table 2. The radiotherapy isodose curves for the treatment of radiotherapy alone are therefore nearly identical to the equivalent radiotherapy isodose curves accounting for the combination of radiotherapy and hyperthermia, with exception for the region inside the outlined GTV where a clear dose escalation is evident. The image with the hyperthermia temperature distribution overlaid clearly shows that the gradual increase in temperature from the dorsal to the ventral side of the GTV is associated with a matching gradual increase in effective radiation dose.

## Discussion

### Effect of Hyperthermia on Radiotherapy dose distribution

This study shows a substantial dose escalation of 7-11Gy when radiotherapy is combined with hyperthermia (Table 3 and Fig. 4). Simulated temperatures match fairly well with the measured temperatures. Radiosensitization is not uniformly distributed, but follows the temperature distribution of the hyperthermia treatment (Fig. 5). As a result, the effective radiotherapy dose is no longer homogeneous within the GTV. The absence of any effect on normal tissue outside the GTV is a result of our assumption that hyperthermic radiosensitization is tumor selective when radiotherapy and hyperthermia are given sequentially with a time interval of 1 h or longer. This assumption is based on preclinical and clinical data. Overgaard determined the effect of sequence and time interval between radiotherapy and hyperthermia on the thermal enhancement ratio (TER) in tumor and normal skin in an in vivo tumor model. He found that the TER in skin equaled that of tumor when radiotherapy and hyperthermia are given simultaneously, but TER in skin quickly decreases to 1 when the time interval is 1 h or longer as currently applied in the clinic [41]. Clinical data for cervical cancer confirm that hyperthermia does not enhance radiation associated toxicity when the time interval between hyperthermia and radiotherapy exceeds 1 h [13].

### Temperature dependence of radiosensitization

The temperature dependence of the LQ-parameters accounted for the temperature dependence of radiosensitization. The large difference between the DVHs based on the linear versus the piecewise-linear interpolation for the temperature dependent *α(T)* and *β(T)* values can be explained by overestimation of the effect of moderate temperature elevations below 41 °C on the LQ-parameters when using a linear interpolation. This overestimation of the effectiveness of hyperthermia results in a very high computed effective dose escalation that is not consistent with the known clinical results for hyperthermia from randomized trials. The dose escalation computed for the piecewise-linear interpolation is more realistic and closer to clinical reality. The piecewise linear interpolation results in a dose escalation that increments much stronger for temperatures above 41 °C, which would imply that temperatures exceeding 41 °C should be pursued in clinical hyperthermia. This demonstrates the need to establish more exact data on the temperature dependent LQ-parameters for the entire clinically relevant hyperthermic temperature range (39–43 °C).

The thermal dose effect relationship used in our simulations is strong and was based on cell survival curves of cervical cancer cell lines [29]. Clinical results for cervical cancer confirm there is a correlation between thermal dose and clinical results. Dinges et al. reported a correlation between local tumor control and CEM43T90 (cumulative equivalent minutes of T90 above 43 °C) for the 4 hyperthermia treatment sessions in a group of 18 patients with advanced carcinomas of the uterine cervix treated with the combination of radiotherapy and hyperthermia (*p* = 0.019) [43]. Franckena et al. analysed treatment outcome for 420 patients with locally advanced cervical cancer treated with radiotherapy and hyperthermia and found a significant correlation between tumor control and survival and CEM43T90 [44]. Franckena et al. found an even more impressive correlation between tumor control and survival and TRISE, a thermal dose parameter based on the product of the median temperature rise and the duration of heating [44].

### Future perspectives

The temperature dependent LQ parameters used are based on in vitro data and represent mainly the direct radiosensitizing effect of hyperthermia on DNA repair pathways [8]. A next step is inclusion of other relevant factors and hyperthermia mechanisms like tissue reoxygenation, direct tumor cell kill, tumor hypoxia and repopulation. Most of these factors can be modelled as changes in the LQ parameters. A recent review discusses how these factors and mechanisms can be incorporated [45].

We did not combine brachytherapy with hyperthermia as combination with the individual 24 PDR pulses given in a period of 24 h is technically impossible. The synergy of hyperthermia given before or after the boost would be limited to a small fraction of the boost. It was therefore sufficient to determine the dose escalation of the EBRT only to estimate the dose escalation of the full radiation treatment due to hyperthermia. Inclusion of the brachytherapy boost in the simulation is logical when an HDR brachytherapy boost is given and combined with hyperthermia, although this is not straightforward. Matching the tissue deformations of the brachytherapy planning MRI images with the external beam radiotherapy CT planning scans is a very challenging task and will require a precise form of deformable image matching. Because of the steep gradients in the brachy dose distribution small errors in geometry due to the matching procedure would lead to relatively large deviations in the brachy dose distribution reconstructed in the EBRT scan. The most robust solution minimizing the risk of introducing errors might be to match the EBRT and hyperthermia datasets onto the brachytherapy image set as the dose gradients in the latter surpass dose gradients in the other sets.

An important step is to move from evaluation to optimization of the combined treatment. The present model evaluated the combined effect of hyperthermia and radiotherapy, where both modalities were individually optimized without taking advantage of the synergistic and complementary effects. In the second step the combined treatment could be optimized. This may result in higher hyperthermia doses being used close to organs at risk with regard to radiotherapy.

The change in Tumor Control Probability (TCP) as a result of adding hyperthermia would be an interesting parameter to analyse. Computation of TCP was left out of the present analysis as the brachytherapy given was not included in the current workflow. For tumors treated by EBRT only, conventional TCP models for radiotherapy [46–48] can be used to calculate TCP based on the equivalent radiation dose. Okunieff et al [47] estimate that TCP increases with 1–3 %/Gy near TCD50 based on cervical cancer treatment data of Perez et al [49, 50]. This corresponds to a 10–30 % increase in TCP due to hyperthermia based on the median dose escalation of 10Gy predicted for our 3 patients (Table 3). This number is in line with the clinical data of cervical cancer trials, e.g. the 26 % increase in tumor control by adding hyperthermia to radiotherapy reported by van der Zee et al. [13].

A very useful application of this model is to compare the effectiveness of different treatment schedules and to guide the design of clinical studies on dose escalation using hyperthermia in a multi-modality setting.

## Conclusions

This study demonstrated that radiosensitization by hyperthermia can be converted to radiation-dose escalation for cervical cancer patients using biological modelling.
